# Characterization of functional traits with focus on udder health in heifers with divergent paternally inherited haplotypes on BTA18

**DOI:** 10.1186/s12917-019-1988-4

**Published:** 2019-07-11

**Authors:** A. Heimes, J. Brodhagen, R. Weikard, H. M. Hammon, M. M. Meyerholz, W. Petzl, H. Zerbe, S. Engelmann, M. Schmicke, M. Hoedemaker, H.-J. Schuberth, C. Kühn

**Affiliations:** 1 Leibniz Institute for Farm Animal Biology (FBN), Institute of Genome Biology, Wilhelm-Stahl-Allee 2, 18196 Dummerstorf, Germany; 2 Leibniz Institute for Farm Animal Biology (FBN), Institute of Nutritional Physiology, Wilhelm-Stahl-Allee 2, 18196 Dummerstorf, Germany; 3 Clinic for Ruminants with Ambulatory and Herd Health Services, Centre for Clinical Veterinary Medicine, Ludwig-Maximilians-University Munich, Sonnenstr. 16, 85764 Oberschleißheim, Germany; 4 Institute for Microbiology, Technical University Braunschweig, Postfach 3329, 38023 Braunschweig, Germany; 5 Microbial Proteomics, Helmholtz Centre for Infection Research, Inhoffenstraße 7, 38124 Braunschweig, Germany; 6 Clinic for Cattle, University of Veterinary Medicine Hanover, Bischofsholer Damm 15, 30173 Hanover, Germany; 7 Immunology Unit, University of Veterinary Medicine Hanover, Bünteweg 2, Geb. 261, 30559 Hanover, Germany; 8 Agricultural and Environmental Faculty, University Rostock, Justus-von-Liebig-Weg 6, 18059 Rostock, Germany

**Keywords:** Genetic selection, BTA18, Bovine mastitis, Somatic cell score (SCS), Clinical performance

## Abstract

**Background:**

A major challenge in modern medicine and animal husbandry is the issue of antimicrobial resistance. One approach to solving this potential medical hazard is the selection of farm animals with less susceptibility to infectious diseases. Recent advances in functional genome analysis and quantitative genetics have opened the horizon to apply genetic marker information for efficiently identifying animals with preferential predisposition regarding health traits. The current study characterizes functional traits with a focus on udder health in dairy heifers. The animals were selected for having inherited alternative paternal haplotypes for a genomic region on *Bos taurus* chromosome (BTA) 18 genetically associated with divergent susceptibility to longevity and animal health, particularly mastitis.

**Results:**

In the first weeks of lactation, the q heifers which had inherited the unfavorable (q) paternal haplotype displayed a significantly higher number of udder quarters with very low somatic cell count (< 10,000 cells / ml) compared to their paternal half-sib sisters with the favorable (Q) paternal haplotype. This might result in impaired mammary gland sentinel function towards invading pathogens. Furthermore, across the course of the first lactation, there was indication that q half-sib heifers showed higher somatic cell counts, a surrogate trait for udder health, in whole milkings compared to their paternal half-sib sisters with the favorable (Q) paternal haplotype. Moreover, heifers with the haplotype Q had a higher feed intake and higher milk yield compared to those with the q haplotype. Results of this study indicate that differences in milk production and calculated energy balance per se are not the main drivers of the genetically determined differences between the BTA18 Q and q groups of heifers.

**Conclusions:**

The paternally inherited haplotype from a targeted BTA18 genomic region affect somatic cell count in udder quarters during the early *postpartum* period and might also contribute to further aspects of animal’s health and performance traits due to indirect effects on feed intake and metabolism.

**Electronic supplementary material:**

The online version of this article (10.1186/s12917-019-1988-4) contains supplementary material, which is available to authorized users.

## Background

In Germany, 733 metric tons of antibiotics for veterinary medicine were distributed in 2017 [1]. Public opinion is increasingly critical towards the use of antibiotics in farm animals as experts warn against potentially increasing resistance of pathogens against antimicrobial drugs in human and veterinary medicine [2, 3].

The infection and inflammation of the mammary gland (mastitis) is one of the most common infectious diseases in dairy cows [4, 5]. Mastitis not only has severe economic consequences (reduced milk yield, veterinary expenses), but can also seriously damage the general health of the animal if left untreated [6]. Therefore, it is the declared aim of researchers and breeders to rear dairy cows with lower susceptibility to diseases such as mastitis while maintaining the performance level of modern, high-yielding farm animals [7]. In previous linkage and association studies, a genomic region on *Bos taurus* autosome 18 (BTA18) has been identified genetically associated with somatic cell score (SCS) in the German Holstein population [8, 9]. The SCS, calculated from the somatic cell count (SCC, cells per ml milk), indicates an impairment of the udder health, and in contrast to SCC, shows a normal or near-normal distribution in the population [10]. Thus, the SCS has been used as a surrogate for udder health in conventional breeding programs by cattle breeders’ associations in many countries to select for improved udder health [11] due to a genetic correlation between SCS and mastitis incidence of about 0.70 [10]. Whether the SCC can also be too low has been the subject of controversy for years, but a baseline of 20,000 cells per ml milk in early lactation cows is assumed [12]. It is reported that udder quarters below 20,000 cells per ml responded to a LPS challenge with a reduced and delayed recruitment of somatic cells into the milk [13].

There are numerous studies confirming that the telomeric region of BTA18 is associated with variations in functional traits such as health, longevity, and fertility [14–17]. However, neither causal genomic variants nor the physiological mechanisms underlying the differences in genetic predisposition are known in spite of many genetic mapping studies with very powerful designs, high resolution genotyping or even whole genome sequence data and thousands of animals. While knowledge on the causal genomic variants would improve specificity of selection, information on the physiological mechanism is essential to evaluate phenotypic consequences and potential detrimental side effects associated with the haplotype beneficial for somatic cell count in milk.

Therefore, the aim of this study was to obtain indication on the potential causal background of the BTA18 association to SCS by collecting clinical and health parameters by deep clinical phenotyping of half-sib heifer groups having inherited alternative paternal BTA18 haplotypes. Thus, the effects of alternative paternal BTA18 haplotypes on essential factors such as feed intake, milk yield, and susceptibility to diseases are explored and provide insight into potential drivers of phenotypic diversity.

## Results

### SCS, udder quarters with low cell count, and bacteriological analysis of milk samples

#### FBN (Leibniz Institute for Farm Animal Biology, Dummerstorf) cohort

**Fig. 1:**
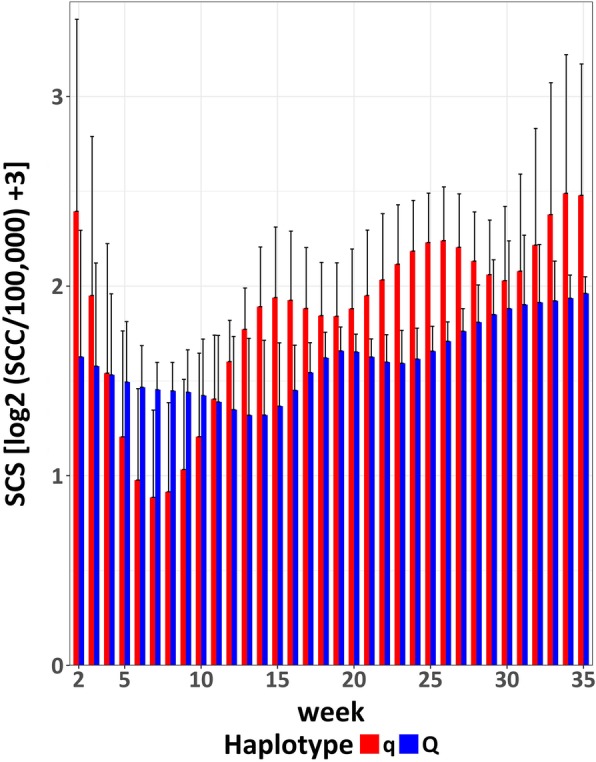
Average weekly somatic cell score (SCS) with standard error across observation period for the Q and q group in the FBN cohort

The average weekly SCS calculated across weeks 2 to 35 was significantly lower for Q animals compared to q animals (lsmean (LSM) = 1.61 (Q) vs. 1.85 (q) log_2_ 1,000 cells / ml, SE = 0.07, *P* < 0.05) as expected based on our hypothesis of the respective haplotype effects. The difference is predominantly due to significant differences between the two haplotype groups (*P* < 0.001) for the interval mid to end of first lactation (week 11 to 35, Fig. 1), with q cows showing a higher SCS (LSM = 2.03 log_2_ 1,000 cells / ml) than Q cows (LSM = 1.66 log_2_ 1,000 cells / ml, SE = 0.07).

**Fig. 2:**
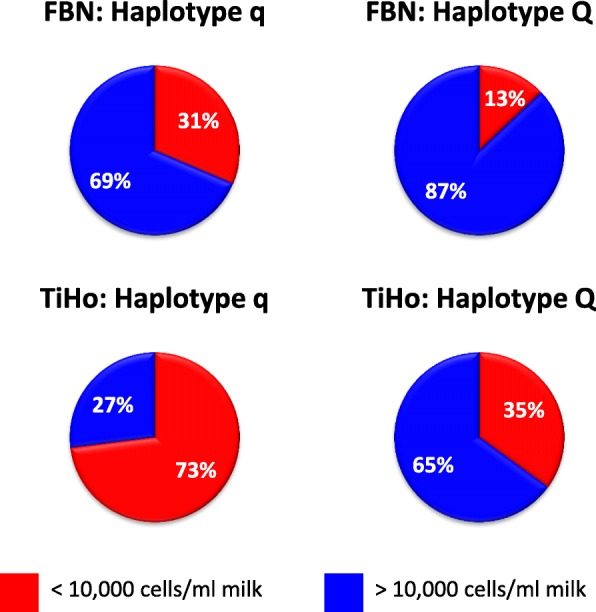
Average weekly somatic cell score (SCS) with standard error across observation period for the Q and q group in the TiHo cohort

In addition to SCS in whole milkings, SCC was determined for each individual udder quarter. In the early lactation period (week 2 to including week 6), a significantly (*P* < 0.05) higher proportion of quarters in the q group (31%) compared to the Q group (13%) was diagnosed as an extremely low somatic cell count quarter (SCC < 10,000 cells / ml, Fig. 2).

The bacteriological analysis of individual udder quarters from the FBN cohort revealed that the colonization of quarters with CNS (coagulase-negative *staphylococci*) was higher for q quarters by trend but not statistically significant between groups (22.4% of all q quarters and 14.3% of all Q quarters), whereas other findings (*streptococci*, *enterobacteria*, *coryneform* bacteria) occurred only sporadically.

#### TiHo (University of Veterinary Medicine Hanover) cohort

**Fig. 3:**
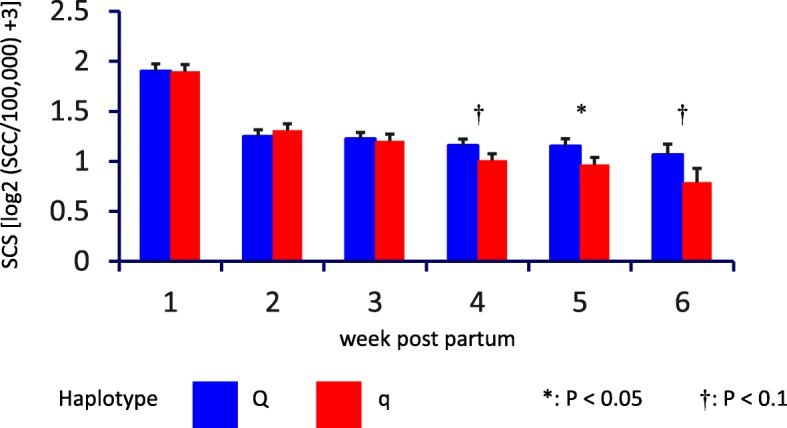
Proportion of udder quarters with extremely low somatic cell count in the Q and q groups for the FBN (week 2 to 6 p.p., difference between Q and q group P < 0.05) and TiHo cohort (week 5 p.p., difference between Q and q group P < 0.01)

For the TiHo cohort, in week 5 after parturition, q heifers displayed a significantly (*P* < 0.05) lower somatic cell score compared to Q animals (Fig. 3), analogous to the numerical differences for the FBN cohort. The differences showed tentative (*P* < 0.1) significance 1 week before and after week 5. For week 6, it has to be considered that only a reduced cohort (*n* = 20) was available, because 16 heifers had already left the experiment at day 36 ± 3 of lactation.

In addition to SCS in whole milkings, SCC for each individual quarter was determined. In the early lactation period, the q group had a higher proportion of udder quarters with extremely low somatic cell count < 10,000 cells / ml milk. This was most prominent in week 5, when 73% of quarters from q heifers, but only 35% of quarters from Q heifers had a somatic cell count below 10,000 cells / ml (*P* < 0.01, Fig. 2). These data are also in line with the observations in the FBN cohort.

### Feed intake, weight, ECM, BCS, BFT, and energy balance in the FBN cohort

**Fig. 4:**
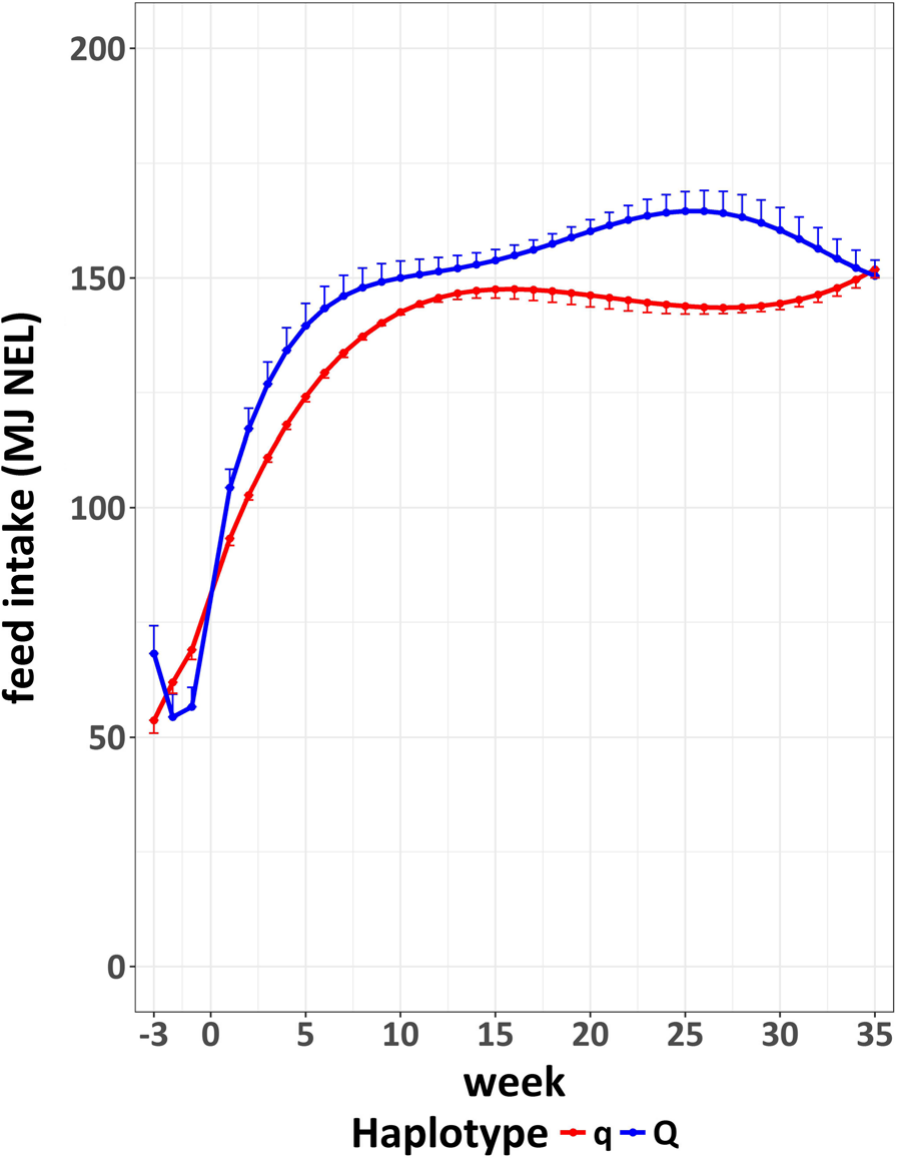
Average daily feed intake within week with standard error across observation period for the Q and q group in the FBN cohort

The average daily feed intake across the entire observation period was significantly different between the two experimental groups at the FBN (*P* < 0.0001, Fig. 4). Over the complete observation period (week 3 a.p. to week 35 p.p.), the average daily feed intake (LSM) for Q animals was 144.5 MJ NEL, for q animals it was 133.3 MJ NEL (SE = 0.50). During the particularly critical phase of lactation, the first 6 weeks after parturition, animals with the haplotype Q displayed a significantly higher average daily intake of energy compared to the haplotype q (LSM = 127.6 (Q) vs. 113.1 (q) MJ NEL, SE = 1.38, *P* < 0.0001).

**Fig. 5:**
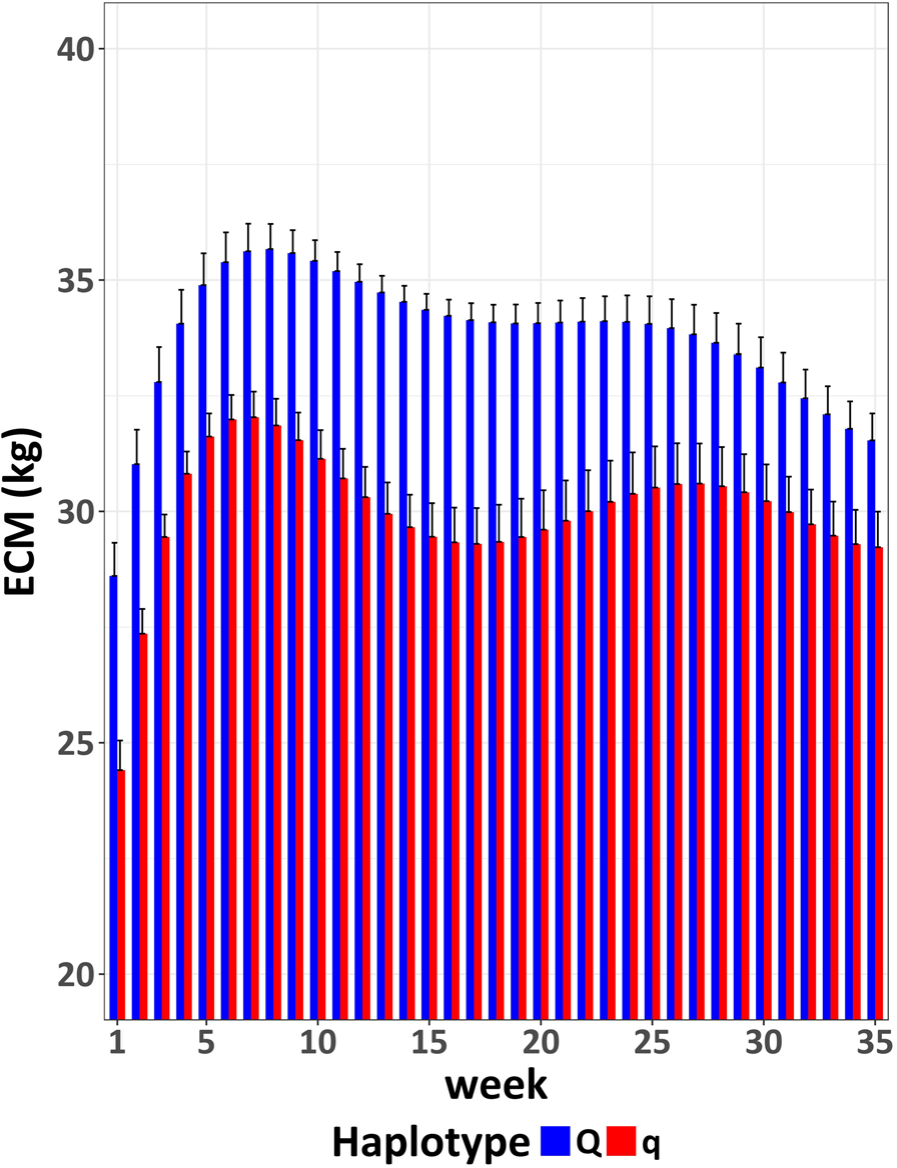
Average daily energy-corrected milk (ECM) with standard error across observation period for the Q and q group in the FBN cohort

The average daily energy-corrected milk (ECM) in the first lactation (week 1 to week 35) was significantly (*P* < 0.0001) higher for Q cows compared to q cows (Fig. 5). The average daily ECM (LSM) was 33.8 kg for Q animals and 30.0 kg for q animals (SE = 0.11). Q cows reached the zenith of milk production in week 8 p.p., q cows in week 7 p.p.

The average body weight (BW) across the entire observation period was significantly different for the divergent haplotypes at the FBN (LSM = 587.5 (Q) vs. 596.1 (q) kg, SE = 0.85, *P* < 0.0001). A time course of the average weekly BW over the first lactation is shown in the supplemental data (see Additional file 1).

The body condition score (BCS) was similar between the two haplotypes at the FBN (LSM = 3.5 (Q) vs. 3.4 (q), SE = 0.03, *P* > 0.1). However, the backfat thickness (BFT) was significantly (*P* < 0.01) different between the divergent haplotypes with the time course shown in the supplemental data (see Additional file 2). The average weekly BFT (LSM) across the observation period was 1.1 cm for Q and 1.3 cm for q cows (SE = 0.04). Whereas the BFT was similar between groups before calving, its *postpartum* decrease was larger in the Q group than in the q group. The lowest BFT was observed in week 16 for both groups.

The calculated average daily EB over the complete lactation was similar between Q and q animals (LSM = 10.9 (Q) vs. 10.1 (q) MJ, SE = 1.08, *P* > 0.1). Either in the particularly critical phase of early lactation (week 1 to including 6 p.p.), there were no significant differences between the haplotypes (LSM = − 9.7 (Q) vs. -13.0 (q) MJ, SE = 3.33, *P* > 0.1), although numerically the q heifers had a more negative energy balance compared to their Q half-sibs.

A time course of the average daily EB over the first lactation is shown in the supplemental data (see Additional file 3).

### Blood parameters in the FBN cohort

When looking at NEFA (non- esterified fatty acids) concentrations in the blood serum, it was noteworthy that two q FBN animals reached values over 1,000 μmol / L a.p.. All three q cows showed a decline in NEFA concentrations before vs. 2 days after calving, but had an increase again and reached the *postpartum* peak of NEFA concentrations 7 or 14 days p.p..

All three Q cows displayed increasing NEFA serum concentrations before vs. 2 days after parturition. The Q cows reached the highest NEFA concentrations between day 2 and 21 p.p.. However, the differences between the divergent haplotypes were not statistically significant, except for day − 10 (*P* < 0.05) (see also Additional file 4). There were no significant differences between the BHB (*beta*-hydroxybutyric acid) serum concentrations of Q and q animals at the FBN (data not shown).

The IGF-I (insulin-like growth factor-I) concentration in blood plasma declined in both experimental groups after parturition. Although the difference between groups was not statistically significant, IGF-I concentration of the Q heifers was numerically higher than their q half-sibs at all time points (see also Additional file 5). The GH (growth hormone) plasma concentrations were similar between the two experimental groups at the FBN (data not shown).

With regard to major blood cell subpopulations, Q cows of the FBN cohort regularly exceeded the reference values for neutrophils (1,000–3,500 cells per μl, indicated by the lab). The two haplotypes of the FBN cohort did not differ in their neutrophil, leukocyte, thrombocyte, monocyte, and erythrocyte numbers in blood (data not shown).

### Health parameters in the FBN cohort

At the FBN, the number of diagnoses resulting in veterinary treatment was higher for q animals than Q animals (q: 18 vs. Q: 14) in the first lactation. An overview of the diagnoses can be found in Table 1.

**Table 1:** Number of diagnoses resulting in veterinary treatment for haplotype Q / q of the FBN cohort

Diagnosis	Haplotype Q	Haplotype q
Retained fetal membranes (> 12 h p.p.)	1	1
Metritis (grade I or II) according to Sheldon et al. [18]	1	1
Clinical mastitis on quarter level according to Hamann et al. [19]	0	1
Subclinical mastitis on quarter level according to Hamann et al. [19]	2	6
Hyperketonemia a.p. BHB ≥ 0.7 mmol / L according to Roberts et al. [20]	0	1
Hyperketonemia p.p. BHB ≥ 1.2 mmol / L according to Roberts et al. [20]	2	0
Orthopedics *Bursitis tarsalis lateralis / praecarpalis purulent/non-purulent, phlegmonia, limax, severe dermatitis digitalis*	7	3
Digestive problems Diarrhea and decreasing feed intake	0	2
Acyclia Ovaries with little or no function	1	2
Bronchopneumonia Disturbed general condition in connection with increased lung noises and increased respiratory rate	0	1

At the FBN, in the 35 weeks p.p., no Q cow had a rectal temperature > 39.5 °C (fever). However, on four different days two q cows had fever. Following the guidelines of Hamann et al. [19], one cow (haplotype q) was diagnosed with a clinical mastitis on one udder quarter in the first lactation. Hamann et al. [19] defined subclinical mastitis by three major conditions: first, one quarter has a SCC > 100,000 / ml; secondly, this diagnosis occurs after day six p.p.; and finally, the bacteriological analysis for this quarter is positive. According to these conditions, two udder quarters of one Q cow and six udder quarters of two q cows developed a subclinical mastitis at different time points in the first lactation. One Q and one q animal each suffered from retained fetal membranes (> 12 h p.p.) and developed a metritis, subsequently (definition and classification according to Sheldon et al. [18]). The Q cow was in treatment for 13 days, the q cow for 28 days, more than twice as long.

Three animals (two q, one Q) at the FBN were treated for acyclia in the first lactation. One of these q cows was under constant veterinary surveillance and was treated multiple times for unresponsive ovaries. Despite great efforts, it was not possible to inseminate this animal successfully for a second lactation. For Q cows, the most common diagnoses were of orthopedic nature (e.g. *dermatitis digitalis*).

## Discussion

Our study confirmed that closely related half-sib heifers, which inherited alternative paternal haplotypes for the targeted regions on BTA18 with presumed effects on health traits, indeed showed significant differences in SCS particularly at udder quarter level during the early *postpartal* period. The numerically lower SCS of q heifers at the beginning of the lactation seems to be contradictory to the initial hypothesis of q animals having an elevated SCC in milk. However, the q heifers displayed a significantly higher proportion of udder quarter samples with an extremely low SCC (< 10,000 cells per ml milk) at the beginning of lactation in both experimental groups kept in different environments. From these data we put up the hypothesis that an appropriate response to mammary infection might be impaired due to the lack of a minimum number of resident protective cells in the milk. In addition to shedded epithelial cells, the somatic cell population in milk comprises leucocytes (including macrophages, neutrophils, and lymphocytes), which are major contributors to the local immune defense [21]. Respective data have been provided describing a low SCC being associated with increased risk for mastitis [22]. Maye et al. [23] could show that milk with a higher SCC more successfully inhibits the growth of a *Escherichia coli* strain compared to milk with low SCC (< 100,000 cells per ml milk). Wellnitz et al. [13] reported a delayed and reduced influx of somatic cells upon LPS challenge in udder quarters with a SCC below 20,000 cells per ml milk. However, in most previous studies, the SCC and SCS were usually considered only at the whole udder level. In pooled samples of all four quarters of the udder, a quarter with an extremely low cell count can be compensated and is therefore not noticeable in routine examinations. Thus, a refined phenotyping is required to conclude on risk status of an animal regarding mastitis.

The Q cows of both cohorts (FBN and TiHo) demonstrated fewer fever days compared to q cows (this paper and Meyerholz et al., under revision). These findings and the increased number of veterinary diagnoses (including subclinical and clinical mastitis) in the q group (both cohorts, Table 1 and Meyerholz et al., under revision) indicate an elevated susceptibility to infectious diseases of q animals compared to Q animals.

In addition to SCC and health recordings, we found further (production) traits that differed significantly between Q and q animals. The Q cows had a higher milk production in terms of ECM compared to the q cows (Fig. 5 and Meyerholz et al., under revision) and also an increased feed intake (Fig. 4). Particularly, the different feed intake should be emphasized, as especially in the first weeks of lactation, the highly lactating dairy cow suffers from reduced energy intake which aggravates the negative energy balance [24]. In the *peripartal* period, the intake of nutrients via feed cannot compete with the required demands for milk production, which forces the dairy cow into a negative energy balance [25]. During this period, several immune mechanisms are modulated and / or suppressed, leading to an increased risk of infectious diseases such as metritis and mastitis [26]. The Q cows, although displaying higher milk yields, were at least as capable of adapting feed intake according to their elevated needs for lactation as the q cows, because the energy balance as calculated from intake and assumed expenditure for milk and maintenance did not differ significantly between the divergent haplotypes. On the contrary, numerically the negative EB of Q in the first weeks after calving was even less pronounced compared to q animals. These data indicate that a potential advantage associated with the BTA18 Q paternal haplotypes regarding disease response as suggested by the lower milk SCS across lactation and lower incidence of veterinary treatment is not due to a lower milk performance and subsequently reduced negative energy balance post partum.

Starting from the same level *prepartum*, the Q heifers had a significantly lower BFT across the course of the observation period due to a higher decline after parturition compared to their q half-sibs. Plasma NEFA is considered an indicator of the degree of fat mobilization from body reserves in response to negative energy balance [20, 27]. The NEFA concentration in blood, however, was significantly higher only at day 10 before calving in heifers with the haplotype q compared to the haplotype Q confirming that differences in energy balance and subsequent fat mobilization are not drivers of the presumed difference in disease susceptibility associated with the targeted BTA18 haplotype.

Rupp et al. [28] identified a point mutation in the SOCS2 (suppressor of cytokine signaling 2) gene which contributes to genetic variance of SCC in sheep. The authors found that the SOCS2 allele, which is considered potentially causal for increased somatic cell count, was also associated with increased milk yield and body weight.

In our study a concordant association of the target haplotype on BTA18 with SCS and body weight was found: q cows displayed a higher SCS as well as elevated body weight. In contrast, the Q haplotype group with superior health traits had a better performance for milk production traits. Thus, the physiological mechanisms underlying the genetic association of the targeted haplotype on BTA18 seem to be different to the recently described causal mutation in the suppressor of cytokine signaling 2 (SOCS2) gene associated with udder health in dairy sheep [28].

GH and IGF-I are main regulators of growth and lactation [29, 30]. The IGF-I in the *periparturial* period sharply declined at parturition and did not elevate back to *prepartum* levels 6 weeks after calving. Essentially, the course of IGF-I concentrations in plasma in the periparturient period was analogous to feed intake and the negative energy balance as discussed by Rhoads et al. [31]. The plasma concentrations of IGF-I were higher in Q compared to q cows (statistically significant in the TiHo cohort (Meyerholz et al., under revision), and numerically for the FBN cohort of the project, see also Additional file 5), but no statistically significant differences in GH before or after calving were observed neither in the FBN nor the TiHo cohort. This confirms previous discussions that during the *peripartal* period of dairy cows, further, yet unknown modulators of the IGF-I level seem to be in action [29].

## Conclusions

This study indicates that the telomeric region of BTA18 harbors a locus, which not only modulates somatic cell count in milk but seems to have additional effects on further traits (e.g. feed intake, disease incidence) in Holstein dairy cows. In addition, there is evidence that this locus influences the pre-infection somatic cell population within individual udder quarters. Due to extremely low somatic cell counts in individual udder quarters early post partum, animals inheriting an unfavorable paternal haplotype in the target region on BTA18 may be at risk for impaired udder health during lactation.

## Methods

### Animals, husbandry, and sample collection

For the study, pregnant, healthy *prepartum* heifers from the German Holstein breed were selected, which addressed a combination of two genomic target regions (43–48 Mb and 53–59 Mb) on BTA18 [32]. The margins of the sub-regions were determined from a previous BTA18 mastitis model (see below).

The first step of the selection process comprised the identification of German Holstein sires with extreme differences for the summarized SNP effects for SCS of their haplotypes in the target regions (see also Additional file 6). For this purpose, initially SNP haplotyping was performed for all individuals within the VIT genome data base for German Holsteins [33]. Genotyping data had been obtained with the 50 k Illumina BovineSNP50 BeadChip (Illumina Inc., San Diego, CA, USA) from routine genomic evaluation for German Holsteins (February 2013 [34]). SNPs were filtered for a minor allele frequency > 1% leaving 43,586 autosomal SNPs for further processing. SNP genotypes were tested for agreement with pedigree information [35]. Only animals with a SNP call rate greater than 98% were further considered. For imputation of missing marker genotypes and for phasing the genotypes, Beagle [36] was used, which relies on population-wide linkage disequilibrium. The UMD3.1 bovine genome assembly [37] served as backbone for all genomic SNP coordinates.

The target regions on BTA18 for our selection process were established based on a previous study [38, 39], in which three sires with confirmed alternative haplotype effects on SCS on BTA18 had been investigated. Inspection of their favorable and unfavorable haplotypes revealed regions common to the three unfavorable haplotypes (q) of those sires. Together with data from the literature [8, 40, 41], the following boundaries of the target genomic regions for this project were defined: rs41880634 (BTA18: 43,098,071) - rs109689271 (BTA18: 47,983,685) and rs29021987 (BTA18: 53,013,208) - rs43072554 (BTA18: 58,696,066). SNP allele effects were summarized within each of the two haplotypes for each sire for the two target intervals and in addition for the region rs41880634 (BTA18: 43,098,071) to the telomeric end of the chromosome. Subsequently, for each of the sires the difference of the summarized effect for its alternative haplotypes was calculated. The following criteria were applied for filtering of sires to be eligible for heifer selection: i) difference in summarized haplotype effect difference at least two standard deviations larger than the mean haplotype difference of all sires for the region rs41880634 (BTA18: 43,098,071) to the telomeric end of the chromosome, ii) difference in summarized haplotype effect difference at least two standard deviations larger than the mean haplotype difference of all sires in at least one of the intervals 43–48 or 53–59 Mb, and finally iii) the sires were not allowed to have inverse phasing regarding the direction of the haplotype differences in the intervals 43–48 or 53–59 Mb.

A total of 156 sires fulfilled these criteria, which were further filtered for age of female offspring at the start of the experiments (at least 18 month of age) and anticipated day of calving (from insemination records to select heifers calving within the experimental time window). Finally, heifers were also submitted to further specific selection steps for sires’ and maternal grandsires’ breeding values for milk performance, overall somatic cell count, milking behavior and for maternal grandsires to obtain high variability within half-sib group, but similar performance level between half-sib groups [32]. Further selection steps comprised the heifers’ predicted age at calving (< 36 months) and the number of potentially available daughters within half-sib group (potentially more than three daughters inheriting the favorable (Q) and three daughters inheriting the unfavorable haplotype (q)).

After genotyping with the 50 k Illumina SNP chip and haplotyping (essentially as described above) the finally filtered 282 heifers were assigned to the Q or q group according to the inherited BTA18 paternal haplotype for the target regions. Those heifers meeting all health and veterinary requirements were purchased from conventional private dairy farms across Germany and allocated about 6 weeks prior to first calving either to the animal experimental unit of the FBN Dummerstorf (*n* = 6, 3 Q, 3 q) for a long-term model or to the Clinic for Cattle at the University of Veterinary Medicine Hanover (TiHo) (*n* = 36, 18 Q, 18 q) for an infection challenge model.

In total, both groups (Q and q) comprised offspring of the same six sires (see also Additional file 6). For the TiHo animals, in each the Q group and in the q group there were a total of five different paternal haplotypes with respect to identical SNP alleles, respectively, because some sires shared identical haplotypes. In the FBN cohort, two of the three paternal q haplotypes and two of the three paternal Q haplotypes were identical regarding SNP alleles.

For the FBN cohort, the experiment was conducted under the reference number 7221.3–1-055/15 with the approval by the responsible authority (LALLF, Landesamt für Landwirtschaft, Lebensmittelsicherheit und Fischerei Mecklenburg-Vorpommern, Rostock, Germany). For the TiHo cohort, the experiment was performed under the reference number 33.12–42502–04-15/2024 by the Lower Saxony Federal State Office for Consumer Protection and Food Safety. Furthermore, this study was submitted to and approved by the ethics committees of the Leibniz Institute for Farm Animal Biology and the University of Veterinary Medicine Hanover, foundation, respectively. All ethical evaluations were performed as required by the German Animal Care law (Tierschutzgesetz, https://www.gesetze-im-internet.de/tierschg/BJNR012770972.html).

The husbandry and sample collection of the TiHo animals was performed as described by Meyerholz et al., under revision. Briefly, all heifers were housed in individual loose stall pens on straw. The animals were milked twice daily, and milk yield was recorded. Weekly quarter milk samples were collected for analysis of milk components, somatic cell count, and microbiological examination. Moreover, weekly quarter milk samples were collected, conserved by bronopol, and analyzed at the MKV Mittelweser e.V. (Milchwirtschaftlicher Kontrollverband Mittelweser e.V., Rehburg-Loccum, Germany) for determination of SCC using the MilkoScan FT Plus (FOSS, Hilleroed, Denmark).

The TiHo heifers were fed one of three component diets (dry period: < 270 days *post insemination* (p.i.), *prepartum* period: > 270 days p.i., and lactation period: after calving). The diets comprised in the dry period hay and minerals, in the *prepartum* period hay, grass silage, corn silage, concentrates, and minerals and in the lactation period grass silage, corn silage, rapeseed extraction meal, soy extraction meal, concentrates, and minerals. The animals left the observation period at day 39 ± 4 after calving.

Six (3 Q, 3 q) heifers were kept in a free-stall barn at the Leibniz Institute for Farm Animal Biology in Dummerstorf (FBN). Husbandry and sample collection at the FBN were performed as follows: Until parturition, cows were housed in calving boxes. After calving, the cows were moved to a dairy cattle loose stall barn and were kept in the same group during the entire observation period. The FBN cows were feed ad libitum with their daily feed intake measured via weighing troughs controlled by the Roughage Intake Control (RIC) system (Insentec, Marknesse, The Netherlands) [42]. The cows were fed different total mixed ratios (TMR) depending on their lactation status (dry: starting at arrival, transit: starting 14 days *ante partum* (a.p.), lactating cows: starting post partum (p.p.)) with adjusted energy content. One representative ratio each for dry, transit, and lactating cows can be found in the supplements of this publication (see Additional file 7). The animals had free access to water.

The FBN cows were milked twice a day in an auto-tandem milking parlor (DeLaval, Tumba, Sweden) with daily recording of milk yield. Moreover, once a week the milk of one afternoon and the following morning milking was pooled and analyzed for content of fat, protein, lactose, urea, and somatic cells at a milk lab (LKV, Landeskontrollverband für Leistungs- und Qualitätsprüfung Mecklenburg-Vorpommern e.V., Güstrow, Germany) using infrared spectroscopy (MilkoScan FT and Fossomatic FC, FOSS, Hilleroed, Denmark). Furthermore, the LKV determined SCC on udder quarter level at specific time points during the lactation (day 2, 7, 14, 21, 28, 35, 42, 70, 150, and 240 p.p.). The SCS was calculated by the following formula: SCS = log_2_ (SCC / 100,000) + 3 [43]. Energy-corrected milk (ECM) was calculated according to Kirchgessner (1997): ECM = average daily milk yield x (0.37 x milk fat percentage + 0.21 x milk protein percentage + 0.95) / 3.1 [9, 44].

The body weight (BW) was recorded daily after the animals were leaving the milking parlor at the FBN. Furthermore, weekly backfat thickness (BFT) was measured by ultrasonic measurement (SonoSite Titan, SonoSite GmbH, Erlangen, Germany) in the sacral region following an established method [45], and simultaneously body condition score (BCS) was assigned according to a standardized scheme [46]. The energy balance (EB) p.p. was calculated with the following formula: EB (MJ NEL) = NEL-intake − (kg ECM × 3.14 + 0.293 × kg BW ^0.75^) [47].

In the observation interval until week 35 p.p. at the FBN, a veterinary clinical examination was performed weekly (daily the first 5 days after calving) to monitor the animals’ health. All veterinary diagnoses and treatments as well as zootechnical interventions (claw care, inseminations etc.) were electronically documented. Rectal temperature was measured daily after the morning milking. In case of infections or diseases, the animals were treated according to good veterinary practice. After first calving, the cows at the FBN were inseminated starting at day 64 ± 23 after parturition, and potential pregnancies were recorded.

Blood was collected by licensed veterinarians from the *Vena jugularis* starting 10 days before the calculated calving date, then 2 days after parturition, followed by weekly sampling until day 42 p.p.. The last sampling days in the lactation were day 70, 150, and 240. The samples from day 10 a.p., 2 p.p., 14 p.p., 70 p.p., 150 p.p., and 240 p.p. were sent to an accredited laboratory (synlab.vet, Berlin, Germany) for differential blood count using flow cytometry and microscope. Serum concentrations of NEFA (non- esterified fatty acids) and BHB (*beta*-hydroxybutyric acid) were determined in samples of day 10 a.p., 2 p.p., 7 p.p., 14 p.p., 21 p.p., and 42 p.p. using the ABX Pentra 400 (HORIBA, Ltd., Kyoto, Japan). Furthermore, plasma samples from the same days were examined for insulin-like growth factor-I (IGF-I) and growth hormone (GH) using validated immunoassays [48].

In addition to samples from whole milkings, quarter milk samples from the FBN cows were taken at the same time points as blood samples and were sent to the lab MQD (Qualitätsprüfungs- und Dienstleistungsgesellschaft Mecklenburg-Vorpommern GmbH, Güstrow, Germany), where in addition to analysis of the SCC a bacteriological status for each udder quarter was determined by qualitative macroscopic evaluation of colonies grown on blood agar.

The cows were killed by immediate exsanguination after stunning with a captive bolt gun (FBN cohort: approximately 6 weeks into their second lactation; TiHo cohort: at day 39 ± 4 after calving).

### Statistical analysis

The data analysis was performed by scripts and packages within the R platform (version 3.4.3) [49]. For the graphical representation of the data, the package ggplot2 was used [50]. To evaluate differences between the Q and q animals, we fitted a linear model to the data using the lm function [51, 52] with fixed effects of group (either Q or q) and week of lactation. For those traits measured daily / weekly across the lactation (feed intake, body weight, ECM, BFT, BCS, and SCS) we fitted orthogonal polynomials or a natural spline to the data to account for missing data points and outliers due to technical problems. For statistical evaluation of a potential significance in different proportions of udder quarters with extremely low somatic cell count and bacterial colonization, a Pearson’s Chi-squared test implemented in the MASS package in R [53] was applied.

## Additional files

Additional file 1: Body weight for FBN cohort: Average body weight within week with standard error across observation period for the Q and q group in the FBN cohort. (JPG 1212 kb)

Additional file 2: Backfat thickness for FBN cohort: Average backfat thickness within week with standard error across observation period for the Q and q group in the FBN cohort. (JPG 1263 kb)

Additional file 3: Energy balance for FBN cohort: Average daily energy balance within week with standard error across observation period for the Q and q group in the FBN cohort. (JPG 821 kb)

Additional file 4: NEFA concentration in blood serum for FBN cohort: Average NEFA concentration in blood serum with standard error at day 10 a.p., 2 p.p., 7 p.p., 14 p.p., 21 p.p., and 42 p.p. for the Q and q group in the FBN cohort. (JPG 704 kb)

Additional file 5: IGF-I concentration in blood plasma for FBN cohort: Average IGF-I concentration in blood plama with standard error at day 10 a.p., 2 p.p., 7 p.p., 14 p.p., 21 p.p., and 42 p.p. for the Q and q group in the FBN cohort. (JPG 667 kb)

Additional file 6: Animal selection process. (PPTX 179 kb)

Additional file 7: Representative TMRs for dry, transit and lactating cows at FBN: Composition of TMRs fed to animals in the FBN cohort during the dry, transit and lactation period. (DOCX 16 kb)

## Data Availability

The datasets used and/or analyzed during the current study are available from the corresponding author on reasonable request.

## Abbreviations

a.p.: *ante partum*
BCS: Body Condition Score
BFT: Backfat Thickness
BHB: *beta*-Hydroxybutyric acid
BTA18: *Bos taurus* Autosome 18
BW: Body Weight
CNS: Coagulase Negative *Staphylococci*
EB: Energy Balance
ECM: Energy-Corrected Milk
FBN: Leibniz Institute for Farm Animal Biology, Dummerstorf, Germany
GH: Growth Hormone
IGF-I: Insulin-like Growth Factor-I
LALLF: Landesamt für Landwirtschaft, Lebensmittelsicherheit und Fischerei Mecklenburg-Vorpommern, Rostock, Germany
LKV: Landeskontrollverband für Leistungs- und Qualitätsprüfung Mecklenburg-Vorpommern e.V., Güstrow, Germany
MKV: Milchwirtschaftlicher Kontrollverband Mittelweser e.V., Rehburg-Loccum, Germany
MQD: Qualitätsprüfungs- und Dienstleistungsgesellschaft Mecklenburg-Vorpommern GmbH, Güstrow, Germany
NEFA: Non Esterified Fatty Acids
NEL: Net Energy Lactation
p.p.: *post partum*
Q: Favorable Haplotype
q: Unfavorable Haplotype
RIC: Roughage Intake Control
SCC: Somatic cell count
SCS: Somatic cell score
SE: Standard error
SOCS2: Suppressor Of Cytokine Signaling 2
TiHo: Clinic for Cattle at the University of Veterinary Medicine Hanover, Hanover, Germany
TMR: Total Mixed Ratio
