# Correction to: Characterization of functional traits with focus on udder health in heifers with divergent paternally inherited haplotypes on BTA18

**DOI:** 10.1186/s12917-019-2034-2

**Published:** 2019-08-08

**Authors:** A. Heimes, J. Brodhagen, R. Weikard, H. M. Hammon, M. M. Meyerholz, W. Petzl, H. Zerbe, S. Engelmann, M. Schmicke, M. Hoedemaker, H.-J. Schuberth, C. Kühn

**Affiliations:** 1 Leibniz Institute for Farm Animal Biology (FBN), Institute of Genome Biology, Wilhelm-Stahl-Allee 2, 18196 Dummerstorf, Germany; 2 Leibniz Institute for Farm Animal Biology (FBN), Institute of Nutritional Physiology, Wilhelm-Stahl-Allee 2, 18196 Dummerstorf, Germany; 3 Clinic for Ruminants with Ambulatory and Herd Health Services, Centre for Clinical Veterinary Medicine, Ludwig-Maximilians-University Munich, Sonnenstr. 16, 85764 Oberschleißheim, Germany; 4 Institute for Microbiology, Technical University Braunschweig, Postfach 3329, 38023 Braunschweig, Germany; 5 Microbial Proteomics, Helmholtz Centre for Infection Research, Inhoffenstraße 7, 38124 Braunschweig, Germany; 6 Clinic for Cattle, University of Veterinary Medicine Hanover, Bischofsholer Damm 15, 30173 Hanover, Germany; 7 Immunology Unit, University of Veterinary Medicine Hanover, Bünteweg 2, Geb. 261, 30559 Hanover, Germany; 8 Agricultural and Environmental Faculty, University Rostock, Justus-von-Liebig-Weg 6, 18059 Rostock, Germany

**Correction to: BMC Vet Res (2019) 15:241**

**https://doi.org/10.1186/s12917-019-1988-4**

The original article [1] contained an error whereby the captions to Figs. 2 and 3 were mistakenly inverted; this has now been corrected.

Furthermore, this error was mistakenly introduced by the production team handling the manuscript and thus, was not the fault of the authors.
