# Supplementary figures and images for: Characterization of functional traits with focus on udder health in heifers with divergent paternally inherited haplotypes on BTA18
Source: BMC Vet Res. 2019 Jul 11;15:241. doi: 10.1186/s12917-019-1988-4 (PMC6624885; doi:10.1186/s12917-019-1988-4)

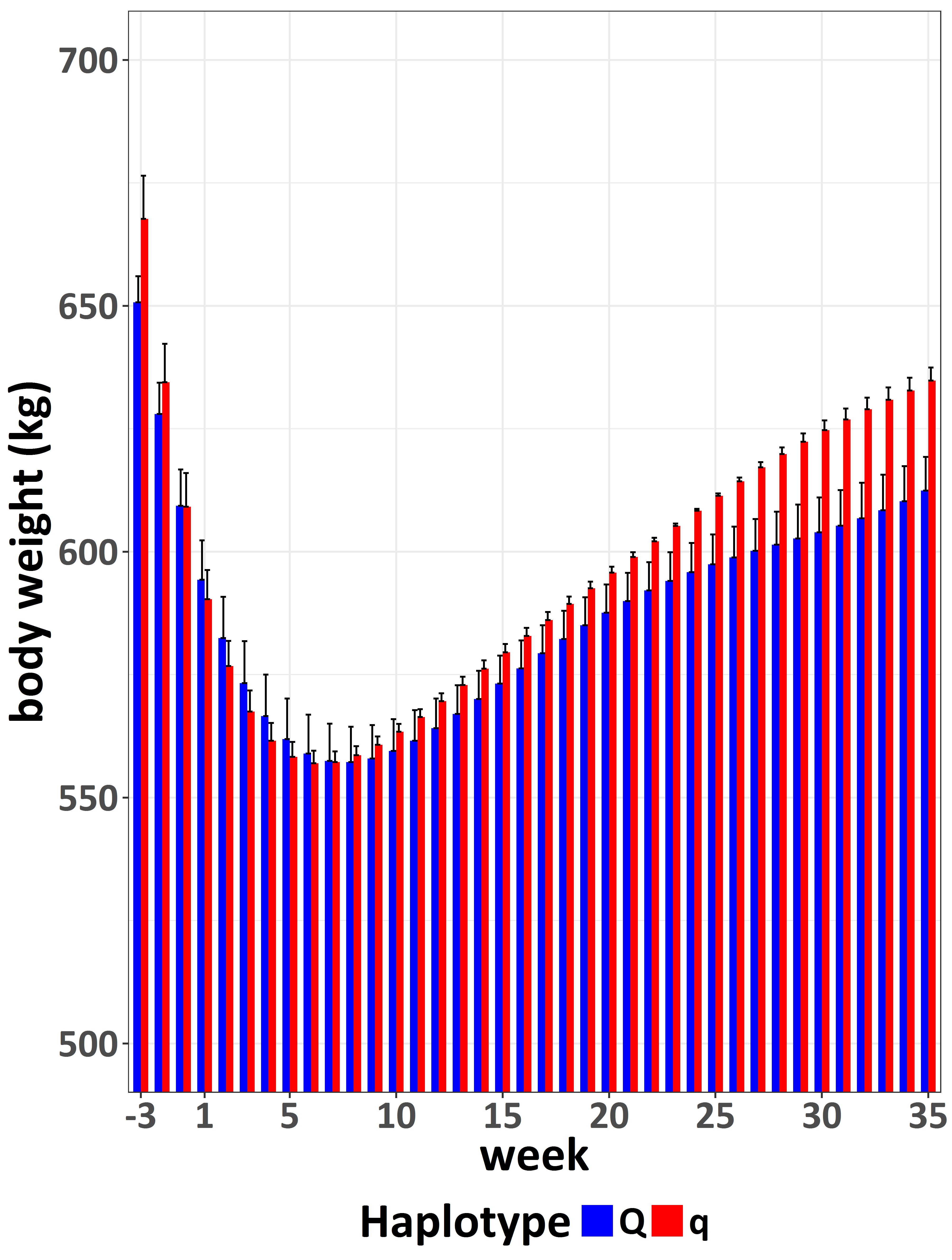

Supplement: Supplementary file 1 — Body weight for FBN cohort: Average body weight within week with standard error across observation period for the Q and q group in the FBN cohort. (JPG 1212 kb) [file 12917_2019_1988_MOESM1_ESM.jpg]

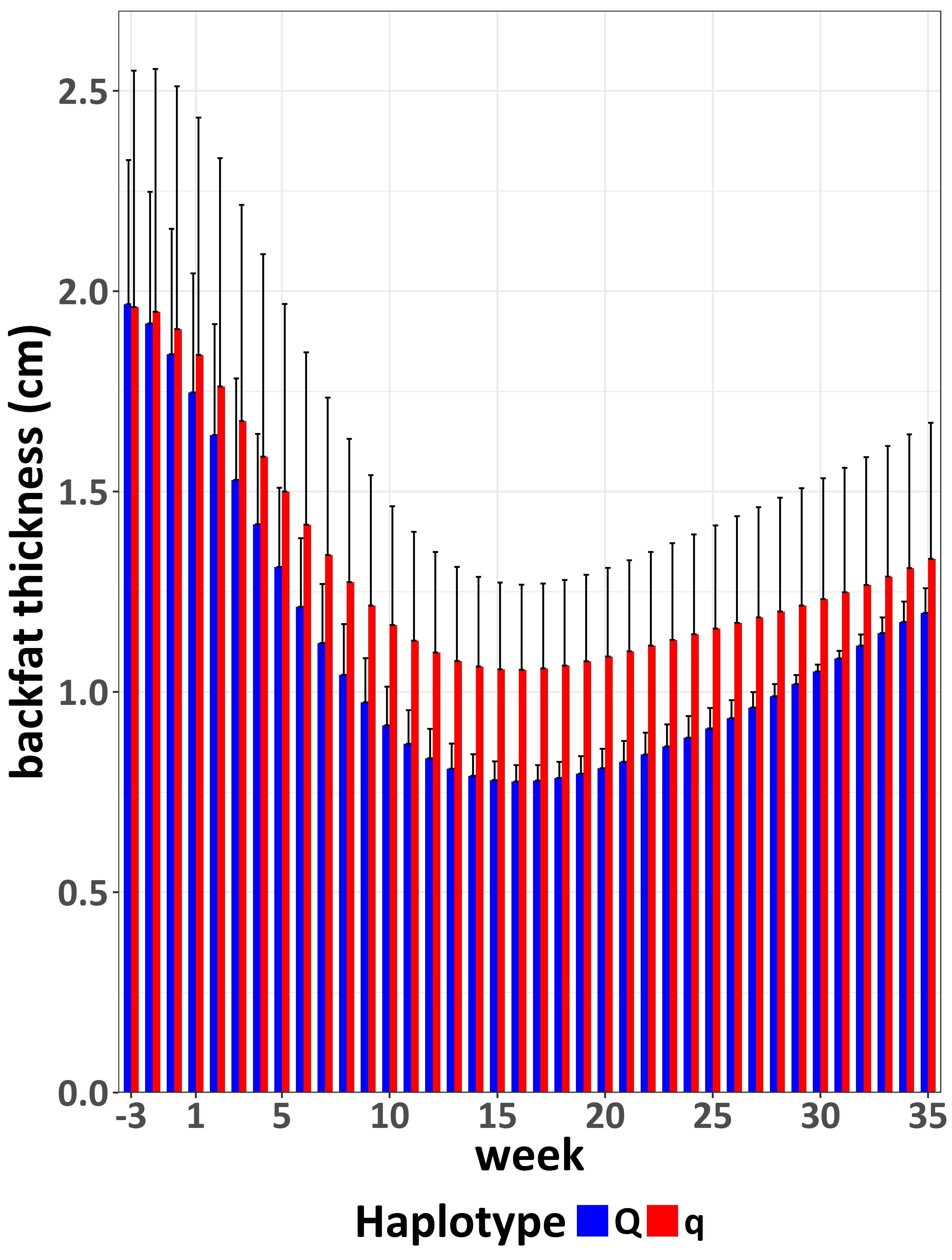

Supplement: Supplementary file 2 — Backfat thickness for FBN cohort: Average backfat thickness within week with standard error across observation period for the Q and q group in the FBN cohort. (JPG 1263 kb) [file 12917_2019_1988_MOESM2_ESM.jpg]

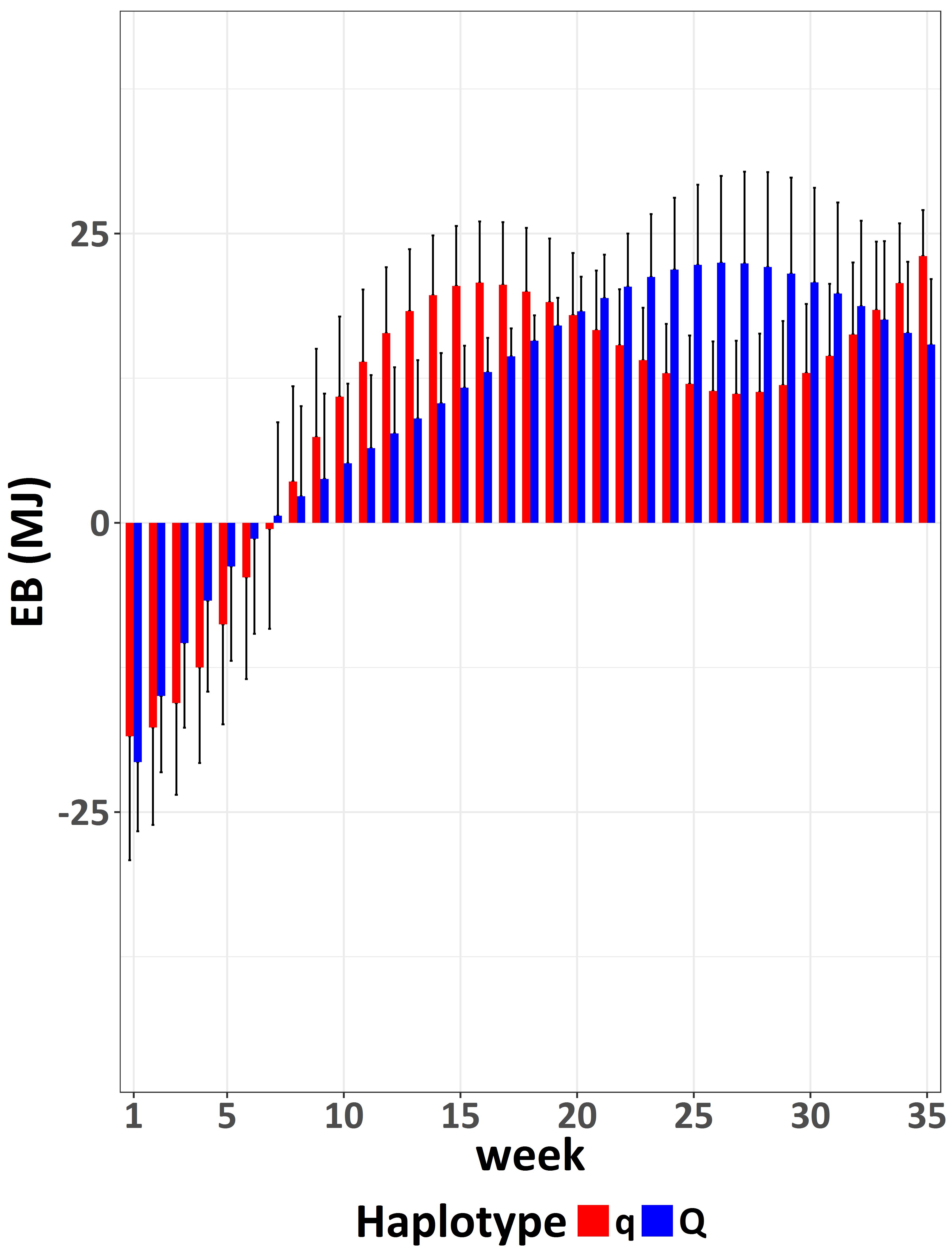

Supplement: Supplementary file 3 — Energy balance for FBN cohort: Average daily energy balance within week with standard error across observation period for the Q and q group in the FBN cohort. (JPG 821 kb) [file 12917_2019_1988_MOESM3_ESM.jpg]

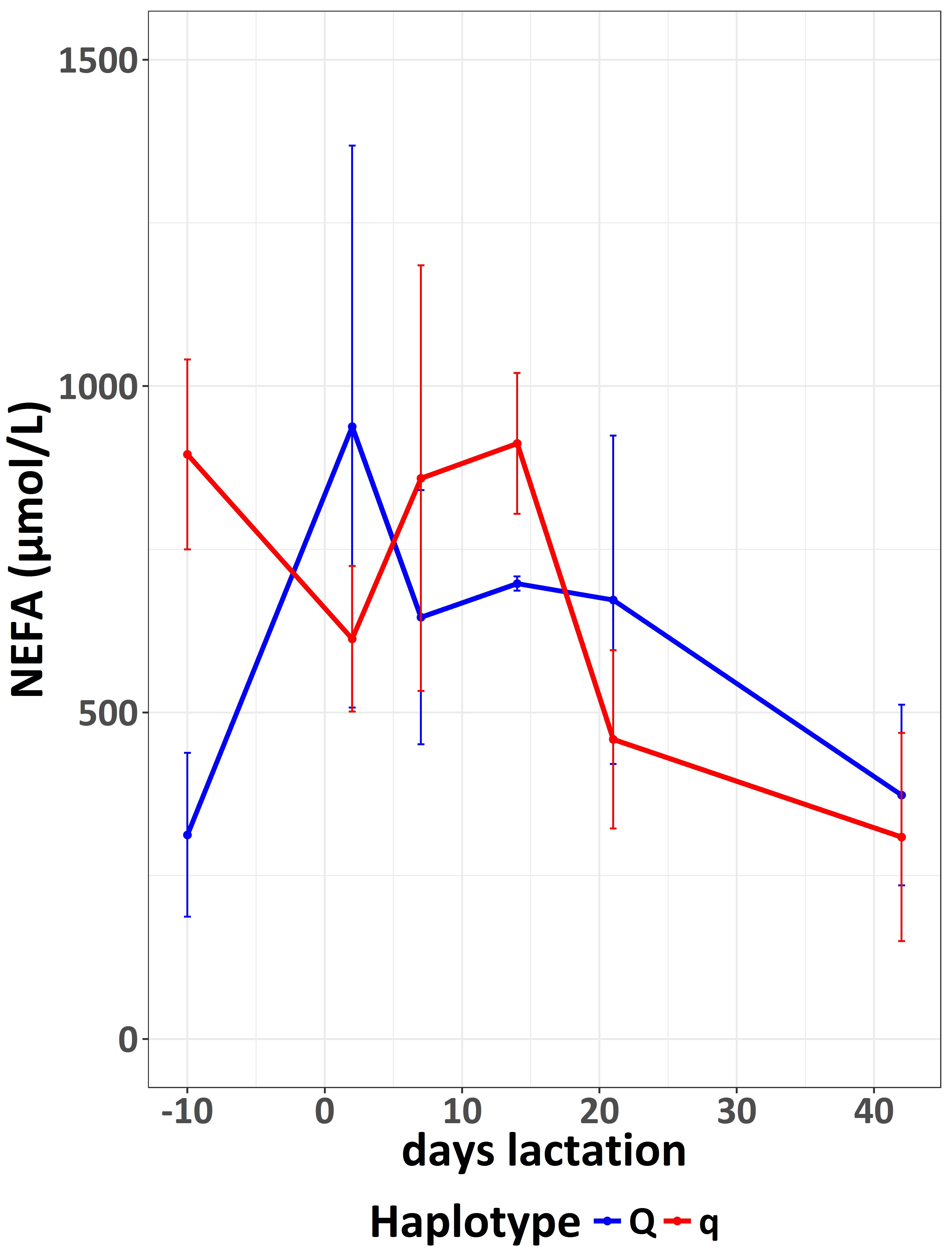

Supplement: Supplementary file 4 — NEFA concentration in blood serum for FBN cohort: Average NEFA concentration in blood serum with standard error at day 10 a.p., 2 p.p., 7 p.p., 14 p.p., 21 p.p., and 42 p.p. for the Q and q group in the FBN cohort. (JPG 704 kb) [file 12917_2019_1988_MOESM4_ESM.jpg]

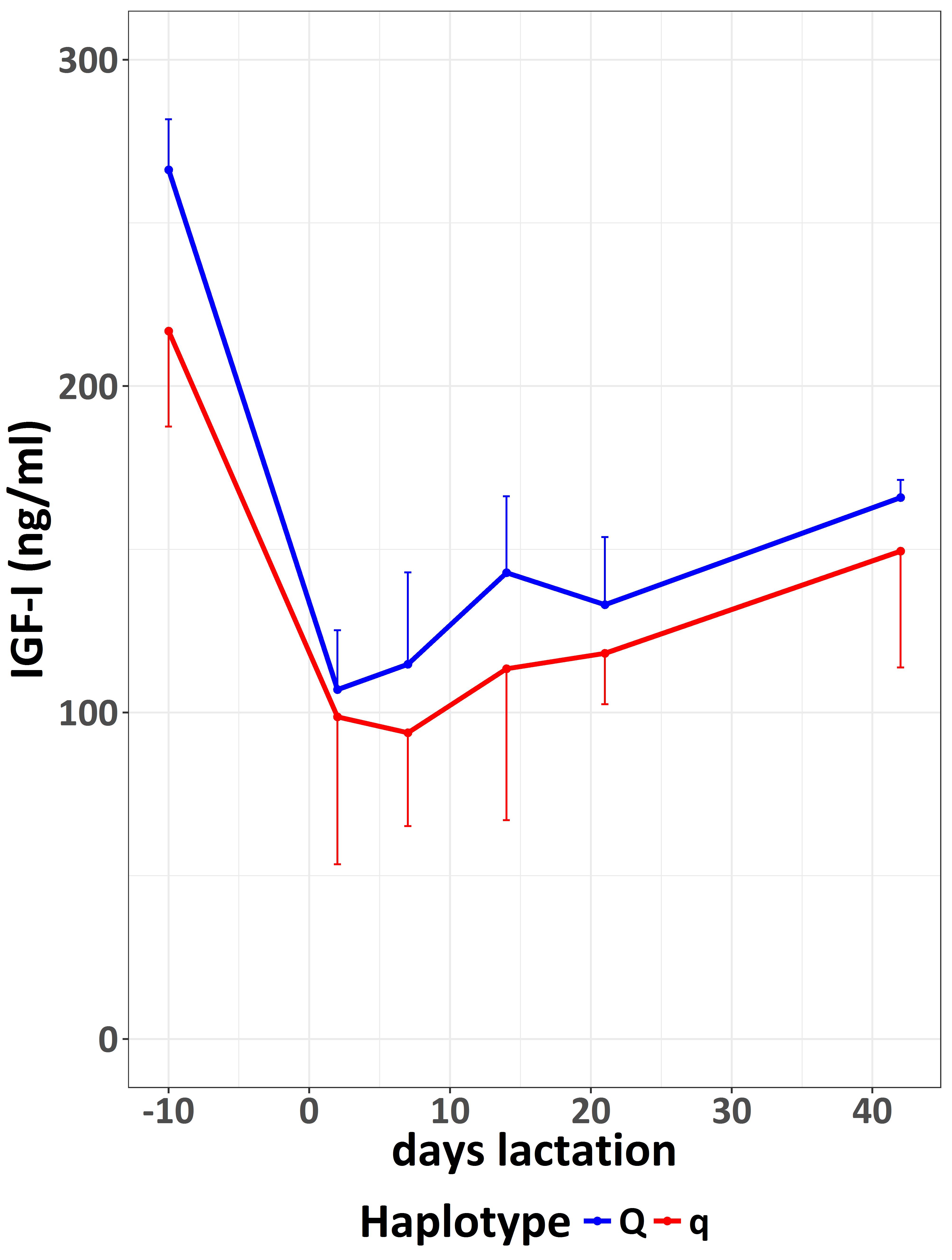

Supplement: Supplementary file 5 — IGF-I concentration in blood plasma for FBN cohort: Average IGF-I concentration in blood plama with standard error at day 10 a.p., 2 p.p., 7 p.p., 14 p.p., 21 p.p., and 42 p.p. for the Q and q group in the FBN cohort. (JPG 667 kb) [file 12917_2019_1988_MOESM5_ESM.jpg]
