# Supplementary material for: Characterization of functional traits with focus on udder health in heifers with divergent paternally inherited haplotypes on BTA18
Source: BMC Vet Res. 2019 Jul 11;15:241. doi: 10.1186/s12917-019-1988-4 (PMC6624885; doi:10.1186/s12917-019-1988-4)
Supplement: Supplementary file 6 — Animal selection process. (PPTX 179 kb) [file 12917_2019_1988_MOESM6_ESM.pptx]

## Slide 1
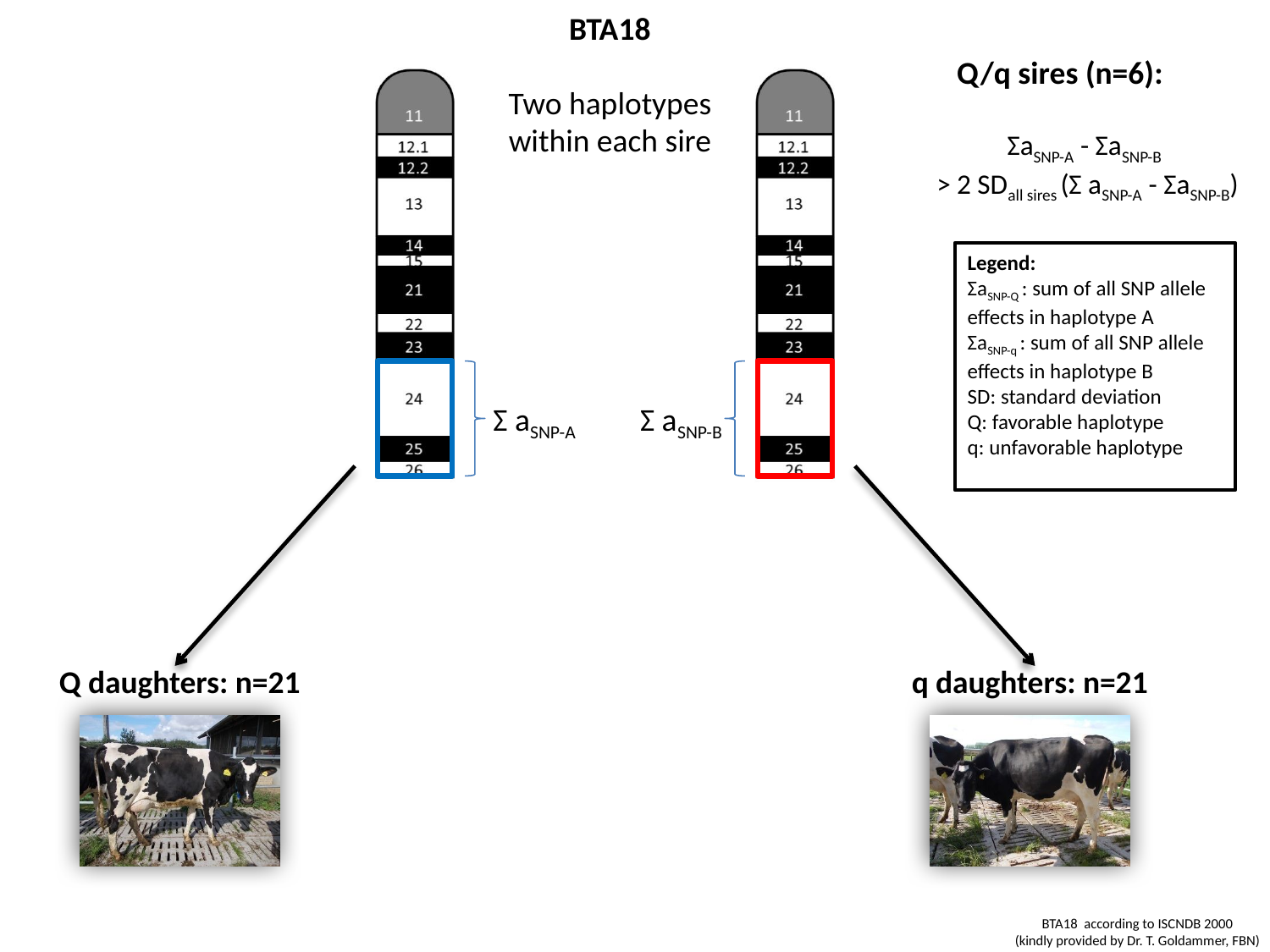

BTA18
Q/q sires (n=6):
Two haplotypes within each sire
ΣaSNP-A - ΣaSNP-B
> 2 SDall sires (Σ aSNP-A - ΣaSNP-B)
Legend:
ΣaSNP-Q : sum of all SNP allele effects in haplotype A
ΣaSNP-q : sum of all SNP allele effects in haplotype B
SD: standard deviation
Q: favorable haplotype
q: unfavorable haplotype
Σ aSNP-A
Σ aSNP-B
Q daughters: n=21
q daughters: n=21
BTA18 according to ISCNDB 2000
(kindly provided by Dr. T. Goldammer, FBN)
