# Supplementary material for: Characterization of functional traits with focus on udder health in heifers with divergent paternally inherited haplotypes on BTA18
Source: BMC Vet Res. 2019 Jul 11;15:241. doi: 10.1186/s12917-019-1988-4 (PMC6624885; doi:10.1186/s12917-019-1988-4)
Supplement: Supplementary file 7 — Representative TMRs for dry, transit and lactating cows at FBN: Composition of TMRs fed to animals in the FBN cohort during the dry, transit and lactation period. (DOCX 16 kb) [file 12917_2019_1988_MOESM7_ESM.docx]

**Representative TMR for dry cows at the FBN:**

| **Components** | **% Dry matter** |
| --- | --- |
| Grass silage | 49.7 |
| Corn silage | 8.3 |
| Hay | 17.2 |
| Straw | 24.1 |
| Mineral 9522* | 0.78 |

Dry matter g/kg fresh matter: 519

Protein g/kg dry matter: 142

NEL MJ/kg dry matter: 5.44

**Representative TMR for transit cows at the FBN:**

| **Components** | **% Dry matter** |
| --- | --- |
| Grass silage | 25.5 |
| Corn silage | 47.5 |
| Hay | 6.6 |
| Straw | 3.3 |
| Rapeseed extraction meal | 3.4 |
| Soy extraction meal | 5.5 |
| MF2000 (pelleted concentrated feed mixture)† | 6.8 |
| Bergophor MFV Plus (mineral feed)§ | 1.5 |

Dry matter g/kg fresh matter: 450

Protein g/kg dry matter: 170

NEL MJ/kg dry matter: 6.58

**Representative TMR for lactating cows at the FBN:**

| **Components** | **% Dry matter** |
| --- | --- |
| Grass silage | 25.4 |
| Corn silage | 41.1 |
| Straw (barley) | 2.48 |
| MF2000 (pelleted concentrated feed mixture)† | 14.5 |
| Corn | 6.7 |
| Rapeseed extraction meal | 4.9 |
| Soy extraction meal | 1.9 |
| Wheat grains | 1.9 |
| Mineral 9522* | 0.72 |
| Feed lime | 0.21 |
| Soybean oil | 0.12 |

Dry matter g/kg fresh matter: 474

Protein g/kg dry matter: 174

NEL MJ/kg dry matter: 6.95

*: Salvana Tiernahrung GmbH, Rosenstraße 9, 25365 Klein Offenseth-Sparrieshoop

†: Ceravis AG Futtermischwerk Karstädt, Postliner Straße 18j, 19357 Karstädt

§: Bergophor-Futtermittelfabrik, 95302 Kulmbach, Betriebsstätte Hohburg Mineralfutter GmbH, Am Lossatal 53, 04808 Lossatal
